# Qualitative exploration of the impact of employment and volunteering upon the health and wellbeing of African refugees settled in regional Australia: a refugee perspective

**DOI:** 10.1186/s12889-018-6328-2

**Published:** 2019-02-01

**Authors:** Nina Wood, Grace Charlwood, Christopher Zecchin, Vibeke Hansen, Michael Douglas, Sabrina Winona Pit

**Affiliations:** 1 Western Sydney University, School of Medicine, University Centre for Rural Health, 62 Uralba Street, PO Box 3074, Lismore, NSW 2480 Australia; 2 University of Sydney, University Centre for Rural Health, Camperdown, NSW Australia

**Keywords:** Refugees, Wellbeing, Employment, Volunteering, Australia, Regional, Volunteering, Work, Social integration, Health care access

## Abstract

**Background:**

People from refugee backgrounds face various challenges after moving to a new country. Successfully securing employment has been linked to positive health outcomes in refugee populations; there is less research into the impact of volunteering on health outcomes in refugees, or the role of employment and volunteering in regional or rural communities. This study aims to explore how employment and volunteering influences the health and wellbeing of refugees settled in regional Australia, and identify areas for appropriate service provision.

**Methods:**

Nine adults of refugee background in regional Australia were purposively sampled through community organisations using word-of-mouth referrals for semi-structured interviews. Interviews were transcribed. Thematic analysis was used to uncover emergent themes and identify relationships between themes. A strengths-based theoretical framework was adopted to inform further analysis.

**Results:**

Paid work and volunteering engenders a sense of self-fulfillment and sense of belonging, facilitating successful integration into a new community. Employment further allows maintenance of an adequate standard of living, thus improving healthcare access and promoting healthy lifestyle behaviours. Adverse effects from employment include difficulties managing work-life balance, disconnect with family and loss of traditional heritage, but these were significantly outweighed by the positive effects. Volunteering provides no financial incentive, but similarly promotes community connections and positive self-worth, preparing refugees for the workforce. Both employment and volunteering held direct positive benefits for their physical and mental health, improved healthcare access and promoted cultural and social integration. These factors enabled successful settlement and subsequently improved overall wellbeing of participants. A strengths-based approach demonstrated how participants used employment as a tool for seeking purpose and ongoing self-development.

**Conclusion:**

Unique experiences with employment and volunteering in a regional area amongst a refugee community were explored. Our results describe various ways in which meaningful employment and volunteering can facilitate positive health and wellbeing outcomes of refugees, and thus reinforces the importance of providing such opportunities to ensure successful settlement. The benefits of volunteering in this community have not been previously explored. Additionally, concerns expressed and recommendations suggested by participants could be used to inform future research, policy, interventions and health and employment service provision for refugee populations.

## Background

People from refugee backgrounds face difficult challenges during the settlement process. Successful integration into a new society is often complicated by issues such as low or no income [1], housing problems [2, 3], language barriers [4, 5], a lack of social support networks and limited access to healthcare [6]. They may experience discrimination in the labour market, and have limited knowledge of the cultural and social nuances of their new community [7]. Issues specific to rural and regional Australia such as remoteness, limited transport options, limited access to health, education and employment services and job opportunities present additional barriers to a positive settlement process and successful integration into their new society [8].

Health and wellbeing is not merely the absence of disease, but results from complex interactions between biological, psychological, social, economic and cultural factors [9]. To form a comprehensive understanding of the health and wellbeing of refugees therefore requires attention to the economic and social issues affecting their lives. Terms such as ‘migrant’ and ‘refugee’ are used differently across the world; for the purposes of this study we adopted the definitions in Table 1 [10].

**Table 1:** Definitions used in this study* [10]

Migrant = generic term for a person moving to another country with the intention of staying for a certain period of time i.e. not a visitor or tourist	
Humanitarian migrant falls under this category = people who have successfully applied for asylum and granted permanent visa status, possibly through private sponsorship	
A **refugee** is one who has a “well-founded fear of being persecuted for reasons of race, religion, nationality, membership of a particular social group or political opinion, is outside the country of his nationality and is unable or, owing to such fear, is unwilling to avail himself of the protection of that country; or who, not having a nationality and being outside the country of his former habitual residence as a result of such events, is unable or, owing to such fear, is unwilling to return to it” (Article 1A(2) of the 1951 Refugee Convention).	

* The terms “refugee” and “humanitarian migrant” are used interchangeably in this study

The Australian government Humanitarian Program provides visas for refugees and humanitarian entrants from overseas [11]. Humanitarian entrants must be supported by a proposer in Australia who takes responsibility for their settlement. Once entrants arrive in Australia through the Humanitarian Program, they are granted permanent residency and can immediately seek employment, access income payments, health and social benefits under the same eligibility criteria as other Australian permanent residents.

Patterns in the backgrounds of refugees who are re-settled fluctuate over time and are influenced strongly by global affairs such as war and other events which trigger mass migration. The Refugee Council of Australia (national umbrella body for refugee organisations in Australia) provides up-to-date advice on current migration issues for the Government to consider when planning the next years’ refugee intake. Refugee intake over the past decade has predominantly included persons of Middle Eastern, South-East Asian and African background [11].

Historically, in Australia, most migrants and refugees have settled in major cities. However, following the Review of Settlement Services in 2004, the policy of the Australian Government has focused on resettling refugees in regional and rural Australia, noting that this strategy was to help “address the demand for less skilled labour in regional economies and to assist humanitarian entrants to achieve early employment” [12]. When applying dispersal criteria to humanitarian entrants, the Australian Government considers presence of relatives or community networks, which often results in formation of settlement populations which are culturally similar [10]. Places in the Humanitarian Program are often taken up by family members of refugees and humanitarian entrants who have already arrived in Australia [11].

Since 2004, the financial support for increasing resettlement in regional areas has increased [13], and between 1996 and 2009, the percentage of refugees settling in regional Australia increased from 5 to 12% [14]. Of the national total, New South Wales (NSW) hosts almost a third of humanitarian entrants to Australia [15], however between 2012 and 2016 only 3.6% of humanitarian arrivals in NSW settled in regional areas [16]. Regional Australia comprises the towns, small cities and areas that lie beyond the major capital cities and comprises around one third of the national population. Major cities, regional areas and remote areas of Australia are differentiated by remoteness scores, derived from measures of road distance between populated localities and service centres [17].

Involvement in vocational activities can be beneficial for the health of refugees in similar ways to other migrant non-refugee populations and members of the general community. Positive health outcomes may include opportunities for social connections, increased meaningful daily activities, developing confidence and providing a sense of purpose [18]. However, there are numerous issues which more specifically affect immigrant populations, such as language and cultural barriers, lack of local experience or understanding of local systems and unmet expectations, which adversely affect the relationship between employment and health and wellbeing [18–20]. Further barriers specific to refugees attempting to join the labour market include the impacts from forced migration, unplanned disruption of previous education and work activities and traumatic experiences. Refugee populations often perform less well in the labour market than other migrant groups with otherwise similar characteristics [10].

Previous studies have explored the interplay between work and health amongst refugee populations. A 2016 report surveying over 300 refugees settled in Australia found that living on low incomes negatively impacted health through financial stress, worry, and creating cost barriers to health-seeking behaviours. Complicating this was the fact that the refugees’ ability to look for work and secure gainful employment was dependent on their physical and mental wellbeing, English proficiency, and confidence levels [21], perpetuating a cycle of health and employment disadvantage.

There is substantial research exploring how factors associated with being of a refugee background influences the ability to secure employment [22–27], and how employment of refugees can have positive economic and social implications for the general community [28, 29]. However, there is considerably less evidence examining the personal impact of being in a regional community and employment on individual refugee’s health and wellbeing.

Additionally, despite there being evidence that volunteering improves elements of health [30, 31], and that there is desire in refugee populations for volunteering opportunities [21], there appears to be little research investigating the perceived impacts of volunteering on health and wellbeing in refugee populations. Thus, the aim of this study was to address this gap in research, and examine how employment or volunteering influences the health and wellbeing of refugees settled in a regional area of New South Wales, Australia.

^*^We acknowledge that after gaining permanent visa status such persons are no longer refugees, but are referred to in this paper as refugees for ease of expression.

### Theoretical framework

To assist in developing novel insights from the data and identify potential areas for practical responses, we adopted a strengths-based perspective as our theoretical framework. Other theories have been used to explore the refugee experience, for example from post-colonial and critical perspectives [32]. As our aim was to explore the contemporaneous experiences and aspirations of participants, rather than on examining any enduring impacts from the past with the attendant risk of re-traumatisation, a strengths-based approach was chosen. A strengths-based approach focuses on the capacities and abilities of individuals to control and develop their own lives in meaningful and sustainable ways [33]. While best known for informing research and therapeutic programs for those recovering from mental illness, a strengths-based approach has been used to guide career development. Kosine et al. [34] described purpose-centred career guidance, which encompassed five main elements in relation to work. Identity, self-efficacy, metacognition, awareness of the two-way relationship between culture and career, and altruistic service represent areas for self-development through recognition of the individuals’ strengths. Work is valued not only for its personal meaning, but also for its broader objective of serving beyond the self, for example the local or global community. A strengths-based approach has previously been adopted to explore various aspects of refugee settlement experiences, including resilience, the role of community refugee organisations and mental health service provision [35–37]. Exploration of refugee experiences often focuses on traumatic past, neglecting refugee strengths, and defining such persons largely as victims [35]. As such, this research aimed to shift perspective from a Western deficits model and explore their experiences in the context of their resilience and ongoing quest for self-development.

## Methods

Semi-structured interviews were used to explore the impact of work and volunteering on individual experiences. The questions were developed from the literature (Table 2) and further refined following review by experts with experience in refugee communities and research design. Following this, an in-depth feedback session was held with a previous refugee to further refine the instrument. The interview was then tested with an initial participant. Based on this feedback and piloting with the first participant, further refinements and modifications were undertaken to develop the final interview questions. (see Table 2).

**Table 2:** Study design

Interview topics	
• Background information (Country of origin, time spent in Australia, working and volunteering status)	
• Relationship between employment, health and health seeking behaviour	
• Relationship between unemployment, health and health seeking behaviour	
• Relationship between volunteering, health and health seeking behaviour	
• Factors facilitating and preventing successful employment and volunteering	
• Areas of need and recommendations	
Inclusion criteria	
• Resettled and currently residing within the local government area	
• Refugee background from overseas	
• At least a conversational level of English	
• Past or present experience working or volunteering in their place of settlement	
• No previous work relationship with the researchers	
Setting^a^	
• Regional area of New South Wales, Australia	
• Major employment industries: health care and social assistance	
• Unemployment rates: ~ 6% in 2016	

^a^ Note: to protect identity of participants no further details of the area are provided

Participants were recruited between July 2016 and March 2017 in regional New South Wales, through local community based refugee and migrant settlement support services. The local refugee community’s composition is in part due to word-of-mouth spread about this region of Australia via refugee camps in Africa, resulting in further applications from those with such connections. To minimize any perceived coercion to participate, participants were first approached by support service staff.

Any interested participants were then contacted by a research team member and provided with information about the study. Participants were also asked if they knew other refugees who could be interested in participating, and asked to engage them and pass on contact details if they agreed. None of the recruiting staff had supervisory or sponsorship relationships with any of the participants.

Participants were further invited through snowball sampling, and purposively sampled for participants with varying levels of employment and volunteering experience, in different work sectors, and for variation in number of years in Australia, age and gender.

Before participating, all participants were provided with written information about the study and signed a consent form. One or two researchers conducted each interview. All researchers were involved in developing the interview questions through iterative discussion at each phase of development and testing, so that all researchers had a shared understanding of the intent of the final questions, the topics to be discussed and how the interviews were to be conducted. Practice interviews between researchers and non-participating volunteers facilitated the process of refining the questions for clarity and sense, and trained the interviewers in guiding the interview.

Interviews were conducted at the public library, participants’ homes or at the university teaching facilities, depending on participant preference. All interviews took place in private rooms to prevent intrusion or distraction and to ensure participant privacy, comfort and anonymity. Each lasted between 45 and 90 min and were audio recorded and transcribed verbatim. Two participants preferred not to be audio recorded, so handwritten notes were taken at the time of interview and transcribed immediately following the interview. Typing of notes was not used during these interviews to reassure participants of privacy, and to maximise interaction and communication between the researchers and participants. Only verbatim interview data was included in the analysis to prevent misrepresentation of the participants’ views.

Interview transcripts underwent thematic analysis to identify, analyse, and report the main themes arising from the experiences and perceptions of the participants [38]. Interview transcripts were read in full by at least two researchers and recurrent experiences and ideas expressed by the participants were identified. These recurring domains were discussed by all research team members and preliminary descriptive codes created to categorise the data according to these codes. Each transcript was then coded by at least two researchers, with codes cross-checked against interview transcripts by a third researcher to ensure coding consistency. Systematic perusal of these descriptive codes was undertaken to generate initial themes. Through iterative review and discussion, researchers agreed on the meanings and developed descriptors for each theme. Conceptually similar themes were then combined into major themes, or differentiated into sub-themes. Ongoing analysis of each new interview content, comparison of the new data to earlier analyses, with either incorporation of the data into existing themes or creation of new codes, ensured all data was appropriately coded and that themes were relevant and captured meaning across the entire data set. Data analysis was conducted concurrently with interviews, with sampling continuing until thematic saturation was achieved, when no more interviews were conducted. The analysis was informed throughout by a strengths-based conceptual framework, but there was no attempt to force the data into pre-defined categories to ensure that our analysis represented the participants’ views and experiences.

## Results

### Sample

Nine participants took part in semi-structured interviews. All were recipients of humanitarian visas, having been sponsored and supported by a local community organization for refugees. They reported working in a variety of jobs in their home country and in Australia, including but not limited to healthcare, media and social services^#^. Volunteer work included unpaid work experience, and charity work with various organisations. Educational qualifications varied from tertiary vocational training diplomas to Bachelor degrees. Limited demographic characteristics are outlined in Table 3 to ensure anonymity.

**Table 3:** Participant Characteristics^b^

Characteristic	Category	Participants (*N* = 9)
Sex	Male	7
	Female	2
Age in years (mean ± SD^a^)	–	38.3 (±10.6)
Years living in Australia (mean ± SD)	–	7.4 (±4.7)
Employed	Yes	6
	No	3
Volunteering	Yes	5
	No	4
Region of origin ^b^	North-eastern Africa	
	West Africa	
Previous employment experience	Yes	5
	No	4

^a^ *SD* standard deviation

^b^ To ensure participant confidentiality, information on participant employment or country of origin has been withheld

**Fig. 1:**
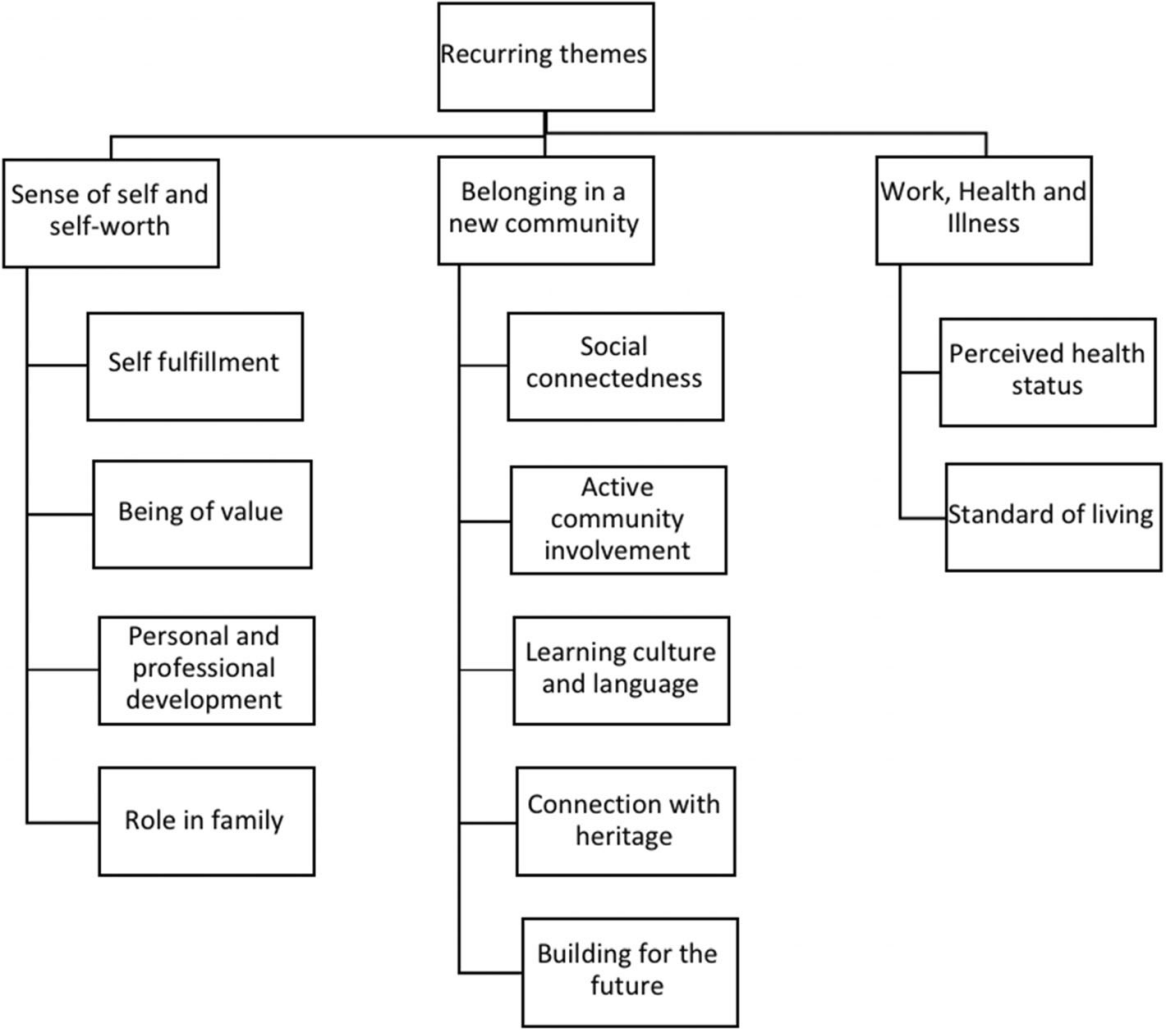
Recurring themes and associated sub-themes

Thematic analysis uncovered three overarching themes and associated sub-themes regarding the perceived impact of participant’s employment and volunteering on their health and wellbeing (Fig. 1):

- Sense of self and self-worth
- Belonging in a new community
- Work, health and illness

## Sense of self and self-worth

### Self-fulfillment

Participants described how employment or volunteering engendered a strong sense of purpose in their lives, and this helped them to feel that they were worthwhile. Successes and focuses at work translated into increased self-esteem, and this was beneficial for their mental health.

*“Makes me to achieve my goal…my goal is to serve humanity and whenever I support clients in achieving a goal, I feel like I have done something good”* (ID07)

However, adjusting to new workplace cultures and ways of work created uncertainties and anxieties, made more acute by the pressure they felt to succeed in their new environment.

*“You feel like, you know…I’m learning, it’s a learning process. And it can make you feel like, you know, I’m not yet ready. (laughs). To take on this big responsibility. Um…yeah, it can be a little bit stressful. You feel like, too much pressure”* (ID01)

Despite the stress of being newly employed, this was viewed to be better than experiencing the low mood and feelings of inadequacy arising from unemployment:

*“Yeah, it’s a shock, because if you don’t have job how can feel. You feel useless. You are useless, you can’t provide anything, even the way how you think. You are thinking negative*” (ID01)

### Being of value

Further to this, many participants explained that employment helped them to fulfil their desire to help others. By helping others at work participants felt they were doing something valuable. Volunteering in particular allowed participants to give back. This in turn affected their self-image and they were able to view themselves in a more positive light.

*“I like helping people… it give me very good feeling that to look after someone”* (ID02*)*

Participants noted how volunteering positively influenced not only their wellbeing, but also enhanced the two-way process of integration, wherein their host community learned from them.

*“With the volunteer I’m doing, I think I’m ok with that and I’ve never experienced something bad in my work. People, they want to know more about me, and maybe they like the way I am, maybe the only African in that community. So they learn a lot of things from me.”* (ID03)

### Personal and professional development

Involvement in the workforce created an ongoing process of learning for participants, through development of their job skills and exposing them to situations for self-reflection and personal growth. Participants valued challenging situations for their learning potential. Rather than being positioned as recipients of pity, such situations were opportunities to be givers of care and to be seen as responsible and conscientious:

*“In fact, it improves my wellbeing. If I go to [place of work] people are in there sick. Put myself in their shoes, try to be empathetic, try to feel the empathy with them, put myself in their position, I could be in this position tomorrow. What can I do? What this person is going through? Always have this empathy not the sympathy…So you see, you get all this encouragement. And even if people criticise me, I try to find out where the criticism come from and how am I going to continue or adjust myself. I want people to criticise me. I mean, if you see me doing something wrong you tell me, I’ll find a way where did I go wrong*”(ID07)

### Role in family

The majority of participants also explained that employment was linked with their perceived role in the family. Particularly for the men, it was important for them to feel that they could provide for their families both in Australia and their home country. Feeling secure in their role as a provider, and thus acting as a positive role model, had a great impact on their self-worth.

*“When you are living with your <siblings>, for you to survive, everyone need to bring something. You put together and you use that together. So when I’m not giving something to the family, it affect my mental wellbeing, and I feel like uneven then”* (ID03)

Both male and female participants commented on the difficulty in balancing the family’s needs with long working hours and study commitments. They outlined the stress that working extended hours created, as well as difficulties associated with less time spent with family.

*“I remember one day, I was around 4 weeks without, having this kind of chat with kids, my <kids>, my family sitting down for meal, like dinner or something like that. And was just feeling very, very disconnected with the family”* (ID04)

## Belonging in a new community

### Social connectedness

Participants explained that employment and volunteering fostered engagement with their new community. These activities afforded participants a substantial degree of social connectedness through the relationships they formed at work with their colleagues, and with the wider community with whom they interacted. As a result, participants felt a sense of acceptance and belonging in their new environment.

*“You are more socialised. You just meeting with people at work. And make, kind of friendship. It just making you feel part of the community. And that is something very important for us. if you don’t have like, close family around here, you feel like…you can just make good friendship at work.”* (ID04)

### Active community involvement

Participants perceived work as a way of contributing to society. For those who could not secure employment, volunteering provided a similar opportunity for desired community involvement. Playing an active role in the community was important not only for their self-perceived value, as described under the theme of ‘sense of self and self-worth’, but also as a way of reciprocating the support given to them in their new communities.

“*You, when you come here, the doors are open for you, you need to come and help to build a window so that other windows will be open for other people. It’s like, what you give back…And that’s why I tell people I’m always very proud of myself, right”* (ID07)

### Learning culture & language

Participants reported that employment and volunteering interacted with culture and language in multiple ways. Their subjectively perceived health and wellbeing were closely linked with ease of integration into their new community. Participants observed that newly settling refugees with limited English struggled to communicate outside of their immediate cultural circles, which consequently diminished their understanding of their new community and confidence and willingness to integrate into the wider community.

“*Socially we had a very good community where you can just go to your friend, go there, eat their food, stay at their house. But their lack of education couldn’t allow you, help you to understand well the system”* (ID04)

Participants also raised concerns about language barriers between themselves and potential employers and work colleagues. Difficulties obtaining employment then negatively affected their ability to learn language immersively, build connections and thus feel a part of the cultural fabric of the community. Even after securing employment, many felt isolated and struggled to build networks in their new work environments.

*“Is quite a hard life. Because you are coming in a country, you don’t know anyone. So you just have to cope with the system. So I was trying just to work, work it out, see how I can cope myself*” (ID08)

Despite concerns regarding the impact of work on disconnection with other aspects of their cultural heritage and past, participants noted that working allowed them to remain mentally active, and thus prevented them dwelling on past traumas.

*“When you are not working you think a lot…you think about your home, you start thinking you know when you hide… I mean when you are just busy keeps you, at least your mind is busy. You can’t think of other things, that’s the same when you are working. No stress” (*ID03)

Participants felt that social interaction at work provided opportunities for developing cultural understanding, which they perceived as fundamental to their integration into the community. As such, those without jobs actively sought volunteering as a means of cultural learning. Despite several participants speaking some English before their arrival, difficulties with local linguistic nuances were encountered.

*“Even when you know the language, you have to understand…because the way we spoke and the way how Australian people spoke is different so because Australian people spoke in shang…shang like slang”* (ID01)

Although frequent discourse with native English speakers at work or volunteering mitigated these difficulties, participants still encountered racial discrimination

*“People say that Australia is good that there is no discrimination but it is. It’s underground discrimination, underground like submarine. I call that submarine discrimination… I was doing my work experience, one employer she was just showing we don’t like you here. She didn’t use the word but the way how the body language is talking directly, send a message we don’t like you here.”* (ID01)

They also noted that cultural differences could lead to mismatched expectations between themselves and their employers.

*“Sometimes in our culture, you can’t call someone and say look, I want to talk about this issue and this issue. Sometimes we want people to come to us, and say” “you alright? Do you need something? I would like to organise a meeting with you, can you come and talk to me about something, about how are you going, and how we can just help you with something”* (ID04)

### Connection with heritage

Although work was a bridge to adopting a new culture, several participants reported that work could lead to a disconnect from their culture of origin. They discussed the difficulties of maintaining their traditional culture and imparting cultural teachings to their children when busy work schedules separated them from their families.

*“As a parent I have to, to balance. I can’t forget my kids, can’t forget my wife… I focus on work, yes I can get money, but here on other side people or my kids just going slowly. Slowly in physical, in mental, in emotional. I have to spend more time with my kids, I have to spend more with my wife and to have debate… to teach them our culture, where I came from…To teach them my culture, and what we do and how we behave and how we dress ourselves.”* (ID01)

Some participants reflected on their own difficult pasts and the ongoing struggles of people in their home countries, but having been accepted in a new country, viewed these struggles as an opportunity to provide meaning and direction in their lives. Volunteering at their local refugee organization, for example, allowed them to give back, created an ongoing connection with their cultural kin in their country of origin and allowed them to exercise their newly established abilities.

*“I feel, all the time, it’s one of my like…my duty. Like I have to do something…no matter what…I have to make sure I’m giving my support to them. Because, just thinking about other people, back in refugee camp. And I know the reality there. Its where I came from… If really, I came from there, and I know the reality…we are few people just coming to this kind of visa, but how many people are still struggling…they don’t have even hope. To come here. And if there is someone who just putting some effort, to help them, we have to do something. That’s something motivating me all the time. To volunteer for [refugee organisation]”* (ID04)

### Building for the future

Employment and volunteering provided foundations upon which participants could build new lives. One third of participants had not secured employment after being in Australia for 3 years. In addition to the ease of cultural integration that working brought, it held immense practical benefits which participants felt helped them in the long term, such as resume building, work experience, and development of skills necessary for workforce participation. Volunteering in particular held such benefits, despite providing no financial incentive.

*“The reason is that is like for me it’s like getting an experience first, to know how the employment force in this country work, the system…to know when I get another job how could myself fit in the workforce”* (ID05)

*“Volunteer, that could have a huge impact on my career…Even if they help you write your resume, and you are going to look for the work where they require for experience, it is still nothing. What I mean, I am trying to say that experience they are requiring, how can I get it?”* (ID03)

## Work, health & illness

### Perceived health status

Employment influenced how participants viewed their health status. They felt healthier overall when working, both from the physical activity of work itself and through providing daily routine and purpose.

*“But if you are moving, something is building in your body and you become active”* (ID01)

*“Doesn’t affect my physical health [negatively]. It gives me more vigour. I’m enthused to go to work*” (ID07)

Conversely, several participants described that work could cause physical strain such as sleep deprivation.

*“Because I was doing 3 job, working 2 jobs and study. So I couldn’t sleep, I couldn’t sleep properly like before”* (ID02)

### Standard of living

Employment was crucial for participants to maintain an adequate standard of living, by enabling them to enact healthy lifestyle behaviours, access healthcare, as well as meet their basic needs. Participants frequently mentioned the desire to avoid poverty, and the role of gainful and meaningful employment in achieving this. The financial insecurity resulting from unemployment, voluntary work or low income was a significant stressor, and had adverse effects upon their psychological wellbeing.

*“That’s a big benefit. You always work for your family, and just make sure. I got to sign for my mortgage, you know? How can I sign it without any permanent position? That’s very, very beneficial, because I know that there is some money will come from there. To pay my mortgage.”* (ID01)

*“Financial stress is a very bad one, because if you are running out of, you don’t have enough income, you are not going to get something to eat”* (ID04)

Gainful employment was universally noted to provide financial security, and nearly all participants noted the subsequent interplay between their financial position and their health. Although none of the participant’s workplaces or employers directly assisted them with accessing healthcare, working allowed participants to partake in healthy lifestyle behaviours, as without the economic means to do so, measures such as buying nutritious food were considered impractical.

*“I don’t have everything I want to have for my physical health and psych health to be good. If I am employed, I can eat what I want. If no work, only cheaper things. And how I can buy medicine?”* (ID09)

Their financial situation affected how they accessed healthcare, including hesitancy at paying for doctors’ appointments and medicine, and needing to visit doctors who bulk billed.**

*“When you are unemployed sometimes you feel like there is something, you need to go and see the doctor. Because you don’t have the money, there is some doctor that say they don’t bulk bill. Because you don’t have the money, you feel like you can just keep that disease in you because it’s not too bad” (ID03)*

**Bulk billing is a payment option under Australia’s universal health insurance scheme (Medicare) wherein certain health services are covered and billed directly to Medicare, resulting in no out-of-pocket cost to the patient.

#### Recommendations to aid in securing employment and access to health care

Participant recommendations were informed by their personal experiences in seeking employment, including the barriers and facilitators they encountered. Practical recommendations and illustrative quotes are displayed in Table 4.

**Table 4:** Recommendations suggested by participants

Individual level	
• Training on how to write resumes, job applications, job interview skills	
• Training and education at work on how to manage stress and poor health, and how to enact healthy lifestyle behaviours	
Simple, collated resources on where to find work and work experience, especially with online accessibilit	
*“I thought to myself I have been an [prior job], I can work anywhere but that’s not how it goes. I don’t know how to build a resume, apply.”* (ID07)	
*“So like more open and simple resources that people can understand so that they know what to look for and where to go” (ID02)*	
*“They should put in place mechanism that will help those who are, English is their second language. Especially to help them with interviews*” (ID08)	
*“More training, like how to manage yourself not to stress out…if you see there is a sign of mental health for the other, don’t leave it long, go to see your doctor. If there is a sign of stress or problem inside, emotion, feeling seek help, seek someone that they can go to nurse, go to GP. That is the way that they increase the knowledge that you can manage your life properly”* (ID02)	
Employer level	
• Culturally sensitive and understanding caseworkers during both settlement process and job seeking process	
• Encouragement or incentives for employers to hire and support settling refugees	
• Opportunities for gaining work experience and training applicable to the Australian workforce	
• Recognition of previous work and educational experience from countries of origin	
*“If they have a case worker, the case worker should be backed with somebody from that person’s background because somebody can come from an English background but it’s not fluent and secondly might be shy.”* (ID08)	
*“Maybe government advocate for us and say okay refugee need to have a safe employment as well, because we can’t do it”* (ID05)	
*“Anyone who is just volunteering, who is looking after a family, should have a kind of…it can be…first assessment. Their first assessment. To know the persons previous work experience. Someone’s previous education level. Someone’s…their ambition of someone. And see all this kind of things, the big picture of the person.”* (ID04)	
System level	
• Increased funding for local refugee support organisations	
• More jobs and employers in regional areas to create more job opportunities	
*“So have to build more companies, to create more jobs, even it can small job but have to open their heart to employ anyone that have experience the job is looking for because most people the job they get is not their own effort it is a connection, like a family connection. So if you don’t have anyone who can connect you to the job you are looking, how can you get a job?”* (ID01)	
*“They [local refugee organisation] really need government support. These guys, they are volunteers. And they put everything together, to settle people, and its…it’s a big task. But they need support”* (ID07)	

## Discussion

Through the lens of a strengths-based approach, our examination of refugees’ experiences with employment and volunteering uncovered the ways in which these vocational activities created purpose. Despite stresses involved in working, and difficulties associated with integration, paid and unpaid work facilitated growth in self-identity, self-efficacy and self-appraisal and ongoing personal and professional development for our participants. Participants valued work that reflected their skills and personal values, and afforded them security and stability, which in turn promoted health-seeking behaviours. Shared cultural backgrounds led to recurring themes about the ways in which participants engaged with work and subsequently with their host community. While their personal experiences of migration and trauma were disruptions to their lives and careers, these experiences also motivated participants to work towards a goal beyond themselves, and serve their local and global community. Using a purpose-centred approach in our analysis of the individual narratives allowed us to focus on outcomes and aspirations as reported by our participants. The framework offers a robust approach to understanding the refugee perspective and for identifying strategies for health promotion and future service provision. Participants did express frustration that their prior qualifications and work experiences were not recognized by potential employers. While this situation may seem counter to a strength-based approach, it also led participants to draw upon and develop personal strengths not restricted by policy or lack of formal documentation such as confidence and resilience, and thus continue their efforts to seek meaningful work, which we discuss below.

### Sense of self and self-worth

Participants’ sense of self-worth and self-esteem were affected by their employment and was closely linked with their psychological health and wellbeing. Participants noted that employment improved self-esteem through a rise in perceived self-worth and sense of personal achievement, as well as through perceived contribution to society and family [23]. A close connection between mental health among refugees and employment issues has been previously outlined [22]. Work is an essential component of individual identity, social roles and status [39], and has been linked to positive self-identity [40].

Challenges at work, and difficulty achieving work-life balance were distressing for participants. Refugees often experience such pressure from balancing multiple household and caring responsibilities [7]. Participants described the pressures of providing for their families in Australia and overseas, and the necessity of employment in facilitating this. Similar findings arose in research on Somali migrant experiences with employment, wherein pressure to undertake extra work was a significant contributor to stress and relationship strains [18]. However, male participants’ self-ascribed roles as providers also gave them purpose, and caused them to feel valued and worthwhile.

Research has found that in populations where prior to resettlement, male refugees occupied traditional roles within hierarchical family structures such as ‘breadwinners’, a perceived loss of this status and change in family roles had negative impacts upon their sense of identity after resettlement [41]. This was noted amongst our participants, who felt pressure to fulfil their brotherly role as a provider. In other Australian research, female refugees who arrive on humanitarian visas have had low levels of engagement in employment, with cultural norms about gendered roles hindering service uptake. This is particularly the case when services do not take into account additional family and care obligations that female entrants may have [42]. Gendered needs for welfare can increase during migration, with prioritization of female carer roles and sexual discrimination in the labour market creating additional barriers to employment, or poor working conditions for those who find employment [43]. While our study did not reveal such differences, further exploratory research may provide insights about the female refugee experience in regional locations.

Unemployment was unanimously associated with disempowerment, and reductions in participant confidence and mood. Employment has been found to be a key determinant for psychological wellbeing, and employment status can reduce odds of major depression [18]. Participants identified unemployment as a significant stressor, supporting research that unemployment is associated with poorer mental health and increased psychological distress [44].

Conversely, as immigrants undertake employment, positive changes in psychological health are seen [45]. Work can be a creative outlet, which may relieve symptoms of depression and anxiety [46], and for our participants, it provided opportunities to remain mentally active and avoid dwelling on past traumas. The impact of pre-migration trauma and settlement-associated stress on mental health has been well documented in the literature [47–51]. However, there is less evidence on employment’s role in alleviating or exacerbating this. Our findings suggest that employment helps participants to avoid re-visiting trauma and grow beyond their pasts through promoting mental activity, creating a sense of purpose and helping establish a new life. In exploring the barriers to meaningful work, participants did not mention regret at leaving their past jobs to come to Australia, nor did they cite their previous histories and associated traumas in our interviews.

### Belonging in a new community

Employment and volunteering allowed participants to forge meaningful relationships and expand their social circles. Research confirms that employment and the job-seeking process increases opportunities for social connections [22, 27, 52], and meaningful employment has been linked to establishing feelings of belonging in newly settling migrants and refugees [23]. Active social participation has been identified as a dimension for measuring successful settlement of immigrants in Australia [53], and a sense of community has been observed to help refugees re-establish their “normal lives” [22]. The desire of participants to “give back” to society was similarly found in previous studies, wherein settling migrants felt obliged to engage with their community and contribute positively to society [7, 29, 54].

Conversely, participants felt isolated when unemployed. Indeed, Carrington et al. [28] found that humanitarian entrants, such as our participants, were often at the greatest risk of social exclusion, as a result of unemployment, reliance on welfare and poverty. Furthermore, refugees holding temporary protection visas rather than permanent visas experience more severe post-migration stresses and this adversely impacts their mental health [55], and further investigation into this sub-population may assist with service development here. Workforce participation can significantly reduce refugee’s social isolation, with consequent positive outcomes for their health and wellbeing [56]. This was a concept with which our participants concurred.

Another cause of stress and poor health in refugees is difficulty adapting to new host community culture [24]. Australia is economically, socially and culturally different from the participant’s home countries, and consequently, cultural dissonance was a recurring experience that affected their settlement. With successful integration, settling individuals often experience loss of cultural norms and connection with their heritage [57]. Participants expressed the desire to maintain such connections with their traditional culture, but faced difficulties doing so.

In spite of language barriers between participants and local community members, involvement in volunteering and work created opportunities for participants to develop their understanding of the English language and Australian culture. They considered this an important part of successful integration into the community. Re-settlement and successful integration is a two-way process, requiring efforts from both refugees and host community. From the refugee perspective, adaptation is needed, but without abandoning their previous cultural identity [11]. Participants experienced racial discrimination at places of work, but also noted that through employment, they were able to help culturally educate community members. Substantial evidence concludes that language and cultural barriers present significant obstacles to refugees securing employment [23]. Given these findings, with appropriate structures in place to assist refugees with securing work, settlement complications arising from language and cultural differences could potentially be mitigated through workforce participation. However, racism and intolerance are important issues to acknowledge and continue to actively address after employment is secured.

### Work, Health and illness

Participants experienced significant stress over their ability to access healthcare without employment and financial security. Gainful employment enabled them to maintain adequate standards of living and “survive”*.* The United Nations High Commissioner for Refugees outlines that *“Economic self-sufficiency is one of the most important factors in successful integration, with earning capacity influencing the ability to ‘purchase’ many of the other resources required to rebuild life in a new country, among them, housing, healthcare and education.”* [58]. Low income presents a barrier to health seeking behaviours [59], and leads to reduced willingness to take time off work to access healthcare [60].

Participants described a re-shifting of their priorities in times of financial uncertainty, during which healthcare needs such as paying for medicine or doctors appointments were forgone in order to meet basic needs, like rent payments. Consistent with these findings, perceived or actual healthcare costs hinder refugee and migrant patients’ access to services [33]. Healthy lifestyle behaviours were also sacrificed, supporting previous research that low incomes prevent refugees from accessing nutritious or culturally appropriate food [24, 61]. Poor nutrition is a contributing factor to declining health status in refugees after arrival [62], and this is an area of concern that requires attention from primary health care providers.

With specific regards to our participants, financial satisfaction has been found as a significant predictor of general life satisfaction in African refugees [27]. Other identified benefits of refugee labour force participation include avoidance of poverty and reduced financial stress [18], and our participants recognised these as protective factors for their psychological wellbeing.

Participants felt healthier when working, and unhealthy when unemployed, regardless of actual illness or whether they engaged in healthy lifestyle behaviours. Consistent physical activity and daily routine from work were seen as fundamental aspects of a normal life, without which they felt incomplete and unwell. Strong links have been drawn between unemployment and poorer physical health outcomes, including increased mortality rates [63]. However, there is conflicting evidence as to whether the converse is true. Although working conveys various benefits for physical health, some aspects of work can pose health risks [64]. In these cases, social context and individual job factors come into play [61]. There is currently a scarcity of evidence examining this impact on physical health or perceived health status in the context of a refugee population, and our findings show the multiple ways in which working exerts positive effects upon health and wellbeing.

### Volunteering

Although there is a relative dearth of empirical evidence concerning unpaid work among refugee communities, this does not necessarily mean that such groups are under-represented in the voluntary sector. Kerr et al. [65] suggest that different cultural perceptions of the idea of a “volunteer” and Western constructs of what constitutes voluntary “work” may discount various voluntary activities that refugees engage in. The culturally nuanced bias of volunteering may call for a different term to be applied to early non-paid, community contribution work.

Participants undertook various types of voluntary work to enhance their community involvement, build their experience and resume, develop understanding of Australian culture and as acts of benevolence.

These included unpaid work experience in their field of choice as well as volunteering at local organisations. Both types of volunteering enhanced community involvement and understanding of host culture, and provided opportunities for self-growth as well as a welcome pastime for those without fulltime employment.

Volunteers of culturally and linguistically diverse backgrounds have previously identified similarly altruistic motivations for volunteering [29]. However, little evidence examines these motivations and benefits in refugee populations, and to our knowledge there is no formal research into this area in Australia, or in the context of regional areas. A 2008 study conducted in the United Kingdom evaluated the impact of volunteering on female refugees, and found that participating in voluntary work allowed the women to participate in community activism, establish identities in relation to their occupational motivations, escape isolation and develop their skills [66]. Volunteering has been proposed as a pathway to employment [29], but there is a need for further research to determine how to establish these mechanisms in Australia.

#### Study strengths

This study involved former refugees with different backgrounds and demographics, allowing us to investigate a variety of lived experiences. The study addressed the impacts of volunteering on refugee health and wellbeing, a previously unexplored topic in research. Furthermore, this relationship was examined within a relatively unexplored geographic context. Both the qualitative nature of our study and the use of semi structured interviews allowed participants to share their experience in depth, in a way that would not have been possible using quantitative methods. Additionally, the interview structure was developed in cooperation with members of the refugee community and support organisations to assess cultural appropriateness.

Although qualitative data involves interpretation of the text, it can be considered trustworthy if it confers credibility, dependability and transferability [67]. To achieve these, our study addressed the intended aims through data collection and analysis that included varied participant experiences, appropriate identification of themes and ongoing dialogue amongst co-researchers to determine agreed-upon data labelling. We systematically reviewed codes and data to develop themes, refining them through iterative return to earlier analyses with each new interview, revising or incorporating new ideas to build a robust and comprehensive analysis of the whole dataset. This process of concurrently and repeatedly collecting and analyzing data until thematic saturation was achieved conferred dependability.

We do not claim that our findings are immediately transferrable to other populations. Nevertheless, our findings were theoretically informed by a strengths-based approach, thus enhancing potential transferability to other groups and settings. The strengths-based approach aligned well with our research aims and participants’ expressed views, in contrast to other conceptual frameworks such as post-colonial perspectives which seek to investigate and acknowledge the ongoing impacts of trauma and history, or community-based intersectoral approaches such as Silove’s ADAPT model [68]. Our analysis aimed to shift away from a deficits-based approach. Interviewers were guided by participant responses, which largely focused on the ways in which employment and settlement experiences had enriched their lives.

#### Study limitations

The restricted area of recruitment limits the representativeness of the findings. A larger study, or one including recruitment in urban areas could have yielded different results. However, in our study, thematic saturation was achieved, with no new themes emerging in the final three interviews. All participants had been sponsored for their visa by a local refugee organisation, affording them a layer of support and financial assistance and sense of permanence. Humanitarian entrants are less likely to be poverty stricken, and may be more highly skilled than other refugees [10], however local employers broadly discount qualifications and work experience from countries of origin, and thus we feel our research still provides necessary insight into the difficulties faced by a marginalized population. Findings may differ for other refugee populations without the same level of security. In those situations, the gravity of employment is of even higher concern and voluntary work may be viewed differently. However, this aspect of our research also allows policy makers to understand the impact local rural refugee support organisations have in regional areas, which may assist in building regional communities and increasing diversity in these areas.

Most participants were male; this is likely to have skewed responses. Despite purposively sampling for maximum variation, we were unable to recruit greater numbers of women to our study. We acknowledge that work and employment has gendered dimensions, although there were minimal differences between the views expressed by our male and female participants. Female participants did not raise gendered issues or note that it had affected their employment. Both male and female participants expressed that family responsibilities created difficulties when navigating work-life balance. Rather than gender not having an influence, our findings may reflect the situation where female entrants join a pre-arrived male family member, and settle into unpaid carer roles within the family. Our study did not include such women as they would not have been eligible as participants, having had no experience with employment. The effect of gender and socioeconomic status on experiences with employment and overall settlement in regional communities is an important area for future research which may allow for better targeted support services for this population.

All participants were of African background, and their acceptance into the pre-established cultural community in the area may have influenced their settlement experiences. This homogeneity is partly due to word-of-mouth spread of knowledge about this region of Australia in refugee camps in Africa. More diverse settlement communities, and those from different cultural backgrounds may face different settlement experiences. The use of English in the interviews may have prevented participants from fully expressing their views, and the interviewers may have been unaware of some cultural sensitivities, as all participants were from non English-speaking backgrounds. Although all were willing to be interviewed in English, this language barrier could have limited recruitment to more confident English speakers. There was limited scope for exploration of the impact of prior employment and qualifications on participant experiences, and this is a potential topic for further research.

#### Implications for future research

This study has demonstrated how employment and volunteering can exert various positive effects on the different aspects of health of refugees in regional Australia. In light of this, our study suggests two areas of potential recommendations for relevant organisations and government bodies, to aid refugees in gaining employment and volunteering opportunities, and consequently promote improved health outcomes. Firstly, there is room for further research into experiences with employment in vulnerable populations not explored in this study, such as asylum seekers, refugees of younger and older ages, those settled in rural or less populated areas, and migrants without sponsorship or guaranteed permanent settlement visas. These populations may experience similar or further disadvantage and marginalisation. Broader examination of their health outcomes could better inform the needs of refugee populations as a whole, and provide foundational evidence for establishing evidence-based frameworks and effective support services in identified areas of need. Sociopolitical factors inform refugees’ experiences [32] and need to be considered in order to provide effective interventions and appropriately tailored services.

Secondly, our participants recommended various strategies for meeting the needs of refugees settling and finding employment, and thus improving their health outcomes. There is a need for research into how to implement more of the practical measures that were identified in the study as facilitators of employment and areas of need.

Similarly, development of strategies or implementation of services to overcome barriers should be an area of investigation. Participants in this study repeatedly acknowledged the local refugee sponsoring organisation as one of the biggest aids, and also commented on the success of local language and job-seeking programs, so these areas could be of particular focus.

## Conclusion

Our findings document various impacts of employment and volunteering upon refugee health and wellbeing. The study revealed the individual experiences and explored personal perspectives of refugees from different backgrounds in regional Australia, providing insights into the varied ways in which engagement in work and volunteering informed their perceived health, wellbeing, and overall self. Few adverse impacts of employment and volunteering were identified, whilst largely positive benefits for physical and mental health, and improved access to healthcare and healthy lifestyles were acknowledged. Positive health outcomes were also mediated by other effects of employment such as facilitation of community involvement and cultural integration, and development of valuable work and life skills. Refugees and other migrants experience similar difficulties in the settlement process, with refugees further experiencing disadvantage stemming from traumatic migratory experiences. This research supports previous knowledge about barriers and facilitators to refugee employment, and discovered novel evidence demonstrating a link between volunteering and positive health outcomes in this population. Even after securing employment, participants encountered difficulties such as racial discrimination, cultural barriers and management of work-life balance. Our research has illustrated the experiences of one group, and raises questions about the transferability of our findings to other disadvantaged communities in other settings. Further such studies, and addressing root causes behind refugee health inequities when designing support structures, may facilitate the implementation of appropriately targeted, effective and sustainable services for refugees.
